# Phenotyping of *ABCA4* Retinopathy by Machine Learning Analysis of Full-Field Electroretinography

**DOI:** 10.1167/tvst.11.9.34

**Published:** 2022-09-30

**Authors:** Sophie L. Glinton, Antonio Calcagni, Watjana Lilaonitkul, Nikolas Pontikos, Sandra Vermeirsch, Gongyu Zhang, Gavin Arno, Siegfried K. Wagner, Michel Michaelides, Pearse A. Keane, Andrew R. Webster, Omar A. Mahroo, Anthony G. Robson

**Affiliations:** 1 Institute of Ophthalmology, University College London, London, UK; 2 Moorfields Eye Hospital NHS Foundation Trust, London, London, UK; 3 Institute of Health Informatics, University College London, London, UK; 4 Health Data Research UK (HDRUK), London, UK

**Keywords:** Stargardt, electroretinography, retina, machine learning, genotype

## Abstract

**Purpose:**

Biallelic pathogenic variants in *ABCA4* are the commonest cause of monogenic retinal disease. The full-field electroretinogram (ERG) quantifies severity of retinal dysfunction. We explored application of machine learning in ERG interpretation and in genotype–phenotype correlations.

**Methods:**

International standard ERGs in 597 cases of *ABCA4* retinopathy were classified into three functional phenotypes by human experts: macular dysfunction alone (group 1), or with additional generalized cone dysfunction (group 2), or both cone and rod dysfunction (group 3). Algorithms were developed for automatic selection and measurement of ERG components and for classification of ERG phenotype. Elastic-net regression was used to quantify severity of specific *ABCA4* variants based on effect on retinal function.

**Results:**

Of the cohort, 57.6%, 7.4%, and 35.0% fell into groups 1, 2, and 3 respectively. Compared with human experts, automated classification showed overall accuracy of 91.8% (SE, 0.169), and 96.7%, 39.3%, and 93.8% for groups 1, 2, and 3. When groups 2 and 3 were combined, the average holdout group accuracy was 93.6% (SE, 0.142). A regression model yielded phenotypic severity scores for the 47 commonest *ABCA4* variants.

**Conclusions:**

This study quantifies prevalence of phenotypic groups based on retinal function in a uniquely large single-center cohort of patients with electrophysiologically characterized *ABCA4* retinopathy and shows applicability of machine learning. Novel regression-based analyses of *ABCA4* variant severity could identify individuals predisposed to severe disease.

**Translational Relevance:**

Machine learning can yield meaningful classifications of ERG data, and data-driven scoring of genetic variants can identify patients likely to benefit most from future therapies.

## Introduction

Inherited retinal diseases are collectively one of the largest causes of blindness in children and working-age adults.^1^ Clinical assessment of patients frequently includes examination of retinal structure using multimodal imaging and assessment of retinal function by electroretinography.^2^ The commonest monogenic retinal disease is associated with biallelic recessive variants in *ABCA4* (Stargardt disease; STGD1; MIM 248200),^3^^,^^4^ with a prevalence of approximately 1 in 8000.^3^ Most patients present with progressive symmetric bilateral central visual loss in childhood or in early adulthood. Classically, a central area of maculopathy develops, surrounded by flecks, and there is usually marked macular dysfunction with or without generalized (mainly peripheral) retinal dysfunction. The latter can be discerned with the full-field electroretinogram (ERG).^2^ The ERG represents the summed electrical response of the retina to flashes of light, delivered under dark-adapted (DA) and light-adapted (LA) conditions, to yield information on the retinal rod and cone systems.

Functional phenotypes have been divided into three groups according to full-field ERGs^5^: dysfunction confined to the macula (normal full-field ERGs; group 1), generalized cone system dysfunction (group 2), or generalized cone and rod system dysfunction (group 3). Several studies have established the prognostic value of this classification in *ABCA4* retinopathy, informing patient counseling and management and potentially influencing the selection of candidates for future interventions.^6^^,^^7^ However, such phenotypic classification requires expertise in ERG interpretation, which is not always widely available.

Another challenge in this condition is the considerable allelic heterogeneity (more than 1000 pathogenic variants have been identified), which confounds precise genotype–phenotype correlation. Nullizygosity (allelic variants that result in a complete loss of *ABCA4* function) is associated with earlier onset and relatively aggressive disease, causing progressive ERG worsening, while a few specific missense variants have been associated with mild disease and normal ERGs.^8^^,^^9^ However, in many cases, it is not straightforward to predict severity of the phenotype (and future prognosis) from the patient's genotype, as the effects of many variants (and their combinations) have not yet been characterized. Machine learning methods are increasingly being explored to assist clinical diagnosis in several fields of medicine, in many cases achieving expert-level performance. Potential advantages include improving efficiency and speed of diagnosis, widening accessibility to diagnosis, developing data-driven approaches to disease classification, and the potential to quickly process historic data or integrate quantitatively across different modalities. At present, there have been relatively few applications of machine learning in relation to visual electrophysiology.^10^^–^^12^ In the current study, we aimed to leverage a uniquely large single-center cohort of genetically and electrophysiologically characterized patients with *ABCA4*-related disease, to help address some of the challenges mentioned above, using machine learning.

We aimed to investigate whether machine learning could be employed to predict ERG phenotypic group from waveform data, which might alleviate problems with access to human expertise. Further, we sought to explore whether a data-driven approach could be taken to score the severity of specific *ABCA4* variants in their effect on generalized retinal function. This could potentially allow identification of those individuals predisposed to more severe disease (disease not restricted to the central retina) based on their genotype. These individuals would be expected to benefit most from early intervention when effective therapies become available. In both investigations of the current study, supervised machine learning methods were used, first to predict labels of ERG phenotypic group (1, 2, or 3) from the waveform data and, second, to predict severity of ERG amplitude reduction from genetic variants.

## Methods

### Inclusion and Exclusion Criteria

All patients included in this retrospective study harbored at least one molecularly confirmed mutation of the *ABCA4* gene and had a clinical presentation consistent with *ABCA4*-related retinopathy. As with other natural history studies of Stargardt disease, those found to have only one pathogenic variant in *ABCA4* but who had a consistent phenotype were included, with the assumption that a second variant was present but undetected. Patients with additional pathology that could potentially influence the ERG were excluded. All had attended the Department of Electrophysiology at Moorfields Eye Hospital between October 2000 and June 2019 and had undergone ERG testing according to the International Society for Clinical Electrophysiology of Vision (ISCEV) standard,^2^^,^^13^ using gold foil corneal recording electrodes. Patients who had undergone recordings with periorbital skin electrodes were excluded. All applicable institutional and governmental regulations were followed (Research Ethics Committee Approval Number 20/HRA/2158). The study adhered to the tenets of the Declaration of Helsinki.

### ERG Data Acquisition and Expert Evaluation

The timing and amplitude of the ISCEV standard DA 10 and LA 3 ERG a- and b-waves and the LA 30 Hz ERG components were quantified and used for comparison with machine learning assessments. The classification into each of the three ERG phenotypes took into account patient age, pupil size during testing, and an assessment of the technical quality of recordings on a scale of 1 (limited compliance/physiologic artifacts but technically adequate recordings) to 5 (highest technical quality). ERG data were acquired at one visit from each patient.

#### Variant Detection

Methods included direct Sanger sequencing, arrayed primer-extension analysis,^14^^,^^15^ testing of multiple-gene panels by next-generation sequencing, and whole-exome or whole-genome sequencing. Patients with pathogenic variants in genes potentially associated with a similar phenotype (chiefly *PRPH2*) were excluded.

#### Data Preprocessing

All data analyses were completed in Python (version 3.6.9) using common libraries for data analysis and machine learning, scikit-learn (version 0.24.2), scipy (version 1.4.1), and numpy (version 1.17.0). Before analysis, individual traces were shifted on the voltage scale by subtraction of the mean of the amplitudes corresponding to the five time point values directly after time point zero (2.5 ms). Individual traces were also interpolated onto a common timeline of 0.5-ms sampling from 0 to 80 ms.

### Automated ERG Trace Selection and Component Labeling

A minimum of two and often many more repetitions of ERG traces were recorded in the cohort data and were used at the time of recording to establish the consistency of responses.^13^ To reduce the influence of artifacts in ERG components, an algorithm was developed for the automatic selection of appropriate traces from a series of repeats, with a maximum of three traces selected per eye per stimulus. For the DA 10 and LA 3 ERGs, selection was according to the minimum mean squared error for three traces within a series of repeats. For the LA 30 Hz flicker ERG, the three traces of maximum 30 Hz signal were selected.

Algorithms were also developed for automated identification of the DA 10 and LA 3 a-wave trough and b-wave peak, as well as of the LA 30 Hz peak. Traces were filtered with a Butterworth filter with an order of 5, then the earliest maxima or minima of traces identified via the first zero-crossing in the first derivative of the filtered trace. A minimum time cutoff was included to prevent the inclusion of artifactual troughs and peaks found within a physiologically improbable time range. The values used for frequency cutoff(s) and time thresholds of the Butterworth filters are detailed in Supplementary Table S1. Mean amplitude values were further averaged across both eyes for the 592 of 597 patients with bilateral recordings. Automatic waveform labeling was validated by an expert (AC) in a random sample of 10% of the data set.

### Classification

**Figure 1.**
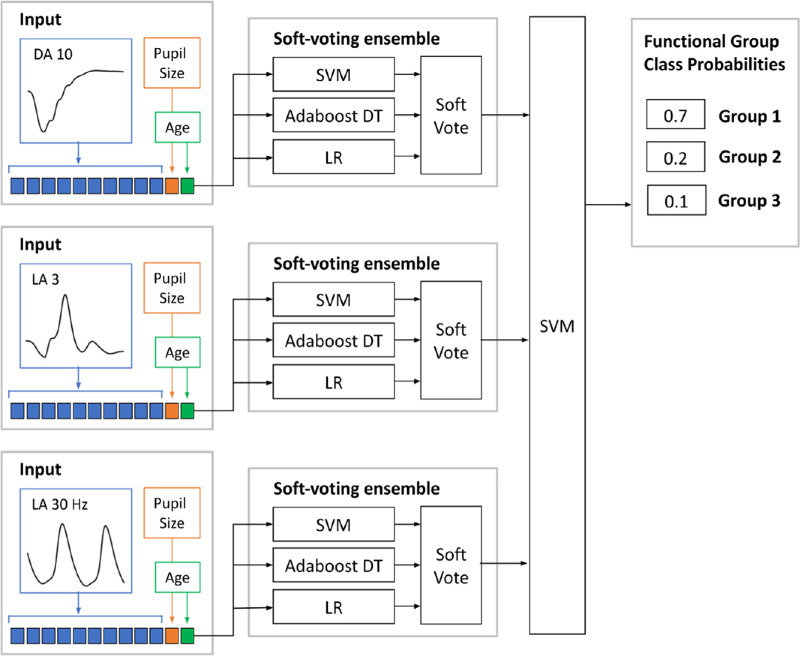
Soft-voting ensemble model for *ABCA4* retinopathy functional phenotype classification with SVMs, Adaboost with decision trees (Adaboost DT), and logistic regression (LR) classification algorithms.

The supervised machine learning pipeline for classification of ERG group is illustrated in Figure 1. Single-trace DA 10, LA 3, and LA 30 Hz ERGs along with patient age and pupil size were used to train a hierarchical soft-voting ensemble model to predict the functional phenotype groups 1, 2, and 3. Ensemble learning is a meta-learning approach that combines decisions from several base learning models to improve the final prediction performance.^16^^,^^17^ This effectively leverages differences in the way the base models are learning predictive features, by allowing each base model to contribute output class probabilities in a vote. The ensemble in this study comprised three base models, including the support vector machine (SVM), adaboost with decision trees, and logistic regression, extended hierarchically in two steps. First, three separate ensemble models were trained using the three base models for traces from each ERG stimulus. Second, the ensembled output class probabilities from step 1 (soft vote) were used to generate functional group predictions per patient using an SVM. Specifically, an average of the output class probabilities for single-trace prediction per eye for each data type was used as input to the final SVM. Within a repeated nested fivefold cross-validation, data were divided at the patient level into training, validation, and test groups (training/validation/test at 64:16:20 split). Tuning of model hyperparameters was performed on the inner validation loop only, and all metrics are reported on the unseen test data set (the remaining 20% data split from each fold) to avoid any information leakage from the test set to the training set.

### Statistical Analysis of Genetic Variants

For investigating the effects of specific genetic variants, β-coefficients were evaluated in an elastic net regression from *ABCA4* variants (with age, sex, and pupil size) to ERG a-wave and b-wave component measurements. The β-coefficients were standardized by *z*-score transformation of the dependent variable. Where one patient had two variants previously identified to occur in *cis* as complex variants, this was assumed to be the case (c.5603A>T, for instance, was included only when not found in *trans* with c.2588G>C p.(Gly863Ala) or c.5461-10T>C). Patients with only one recognized variant or more than two variants were excluded from the regression analysis. The model was evaluated through leave-one-out cross-validation and calculation of the *r*^2^ score on the unseen test group.

### Role of the Funding Source

The funders of the study had no role in study design, data collection, data analysis, data interpretation, or writing this report.

## Results

### Patient Demographics and Genetic and ERG Characteristics

**Figure 2.**
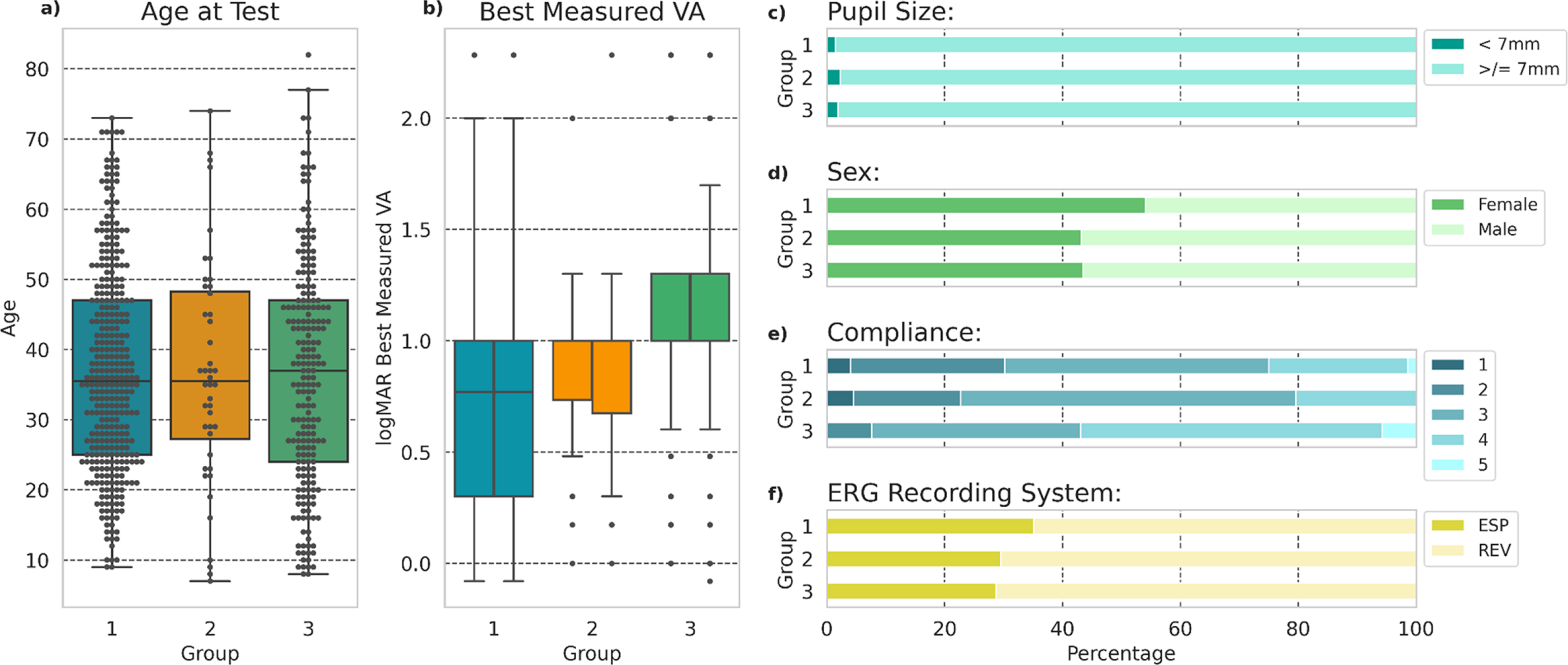
Patient cohort demographics and other baseline parameters by expert-assigned electrophysiologic group. (a) Age at testing. (b) Best-measured visual acuity at testing. (c) Pupil diameter (greater or less than 7 mm). (d) Sex. (e) “Compliance” score (5 denotes highest technical quality recordings). (f) ERG recording system (ESP, LED-based Diagnosys Colordome running Espion software; REV, Xenon flash stimulator and “Observer/Reviewer” software).

ERGs were available from 597 individuals with *ABCA4* retinopathy (1189 eyes), including 497 with at least two recognized pathogenic variants. Prevalence of different variants for the cohort is given in Supplementary Table S2. According to expert (human) analysis of the ERGs, the cohort comprised 344 patients in group 1 (57.6%), 44 in group 2 (7.4%), and 209 in group 3 (35.0%). Variables of age, pupil size, sex, compliance, and ERG recording system were similar for each of the three ERG groups (Figs. 2a, 2c–f). The greatest variability in best-correct visual acuity was seen in those with the group 1 ERG phenotype, with those in groups 2 and 3 having more consistently severe central visual impairment (Fig. 2b).

There was a high degree of interocular symmetry in the main ERG components, including DA 10 ERG a-wave (*r* = .96) and b-wave (*r* = .96), LA 3 ERG a-wave (*r* =.90) and b-wave (*r* = .96), and LA 30 Hz ERG peak amplitudes (*r* = .95). Slope coefficients for the interocular ERG components ranged from 0.99 to 1.00 given intercept 0.

**Figure 3.**
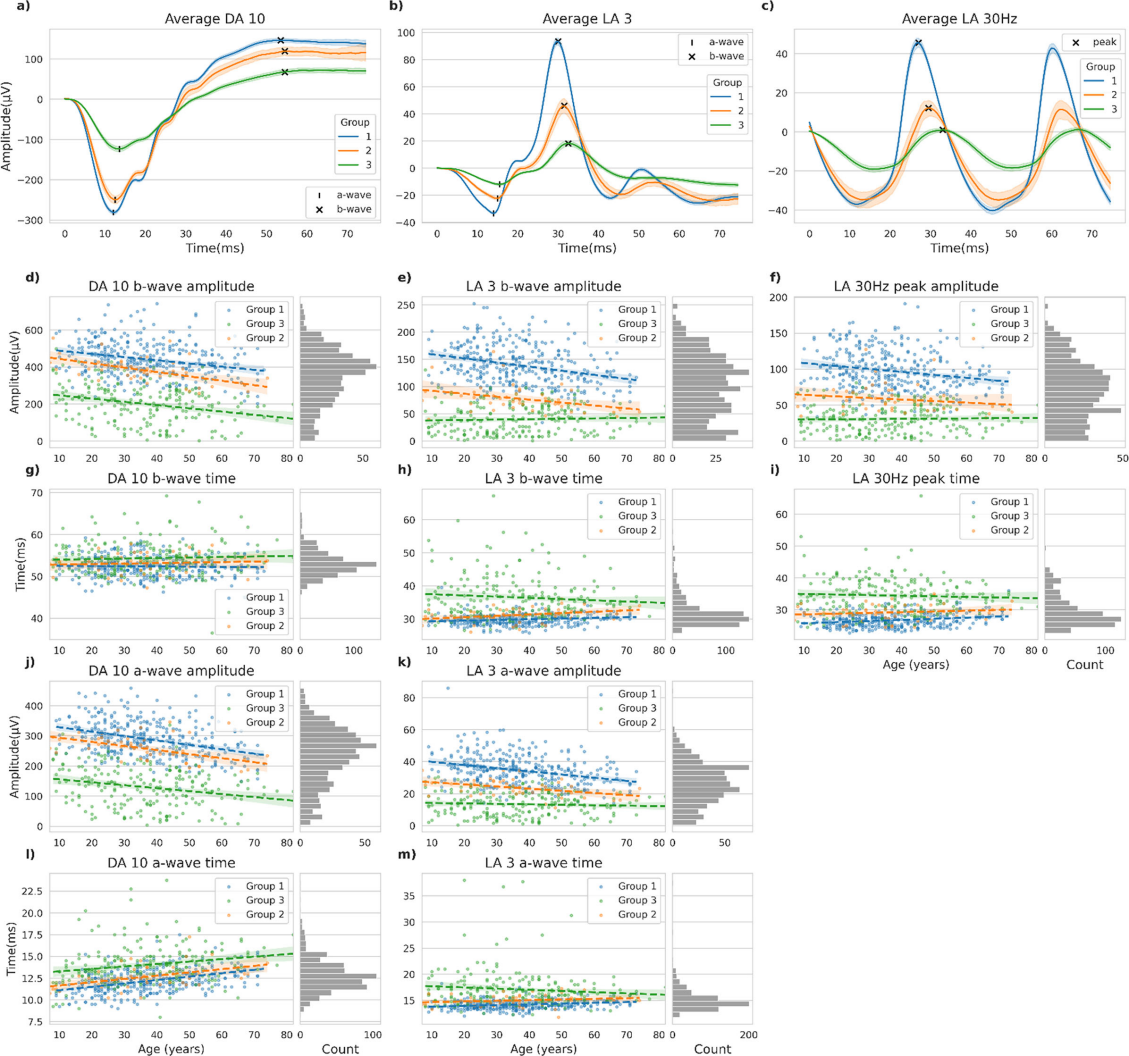
(a–c) Grand average of DA 10, LA 3, and LA 30 Hz ERG traces within each of the three ERG phenotype groups; shaded areas give 95% confidence intervals. (d–m) Amplitudes and peak times of the main ERG components for every patient, plotted against age, and illustrating the data range for each of the three groups; *broken lines* show linear regression lines for each group (*left-hand plots*); histograms illustrate parameter distributions (*right-hand plots*).

Figures 3a–c show grand averages of DA 10, LA 3, and LA 30 Hz ERGs for each of the three expert-assigned groups. Mean amplitudes were lowest and peak times longest for the ERG components in group 3, characterized by both cone and rod system dysfunction. Mean amplitudes were greatest and mean peak times shortest in group 1, with group 2 patients showing intermediate mean values. A comparison of ERG amplitudes with age (Figs. 3d–m) suggested a reduction in all ERG components with increasing age in groups 1 and 2 (Table 1). In group 3, no age-associated reduction was evident in the LA 3 and LA 30 Hz ERGs. All three groups showed greater mean DA 10 ERG a-wave peak times with increasing age. LA 3 and LA 30 Hz ERG peak times showed greatest delays and variability in group 3, but linear regression revealed no evidence of age-associated worsening in this cross-sectional analysis.

**Table 1.** Mean Rates of Change of Amplitude with Increasing Age in Microvolts/y, in ERG Groups 1 to 3 in the Cross-Sectional Data Set

	Rate of ERG Component		
	Amplitude Change (µV/y)		
ERG Component	Group 1	Group 2	Group 3
DA 10 b-wave	−1.72	−2.36	−1.75
LA 3 b-wave	−0.75	−0.54	ND
LA 30 Hz peak amplitude	−0.41	−0.22	ND
DA 10 a-wave	−1.46	−1.35	−0.98
LA 3 a-wave	−0.20	−0.13	ND

ND, no decline detected.

**Figure 4.**
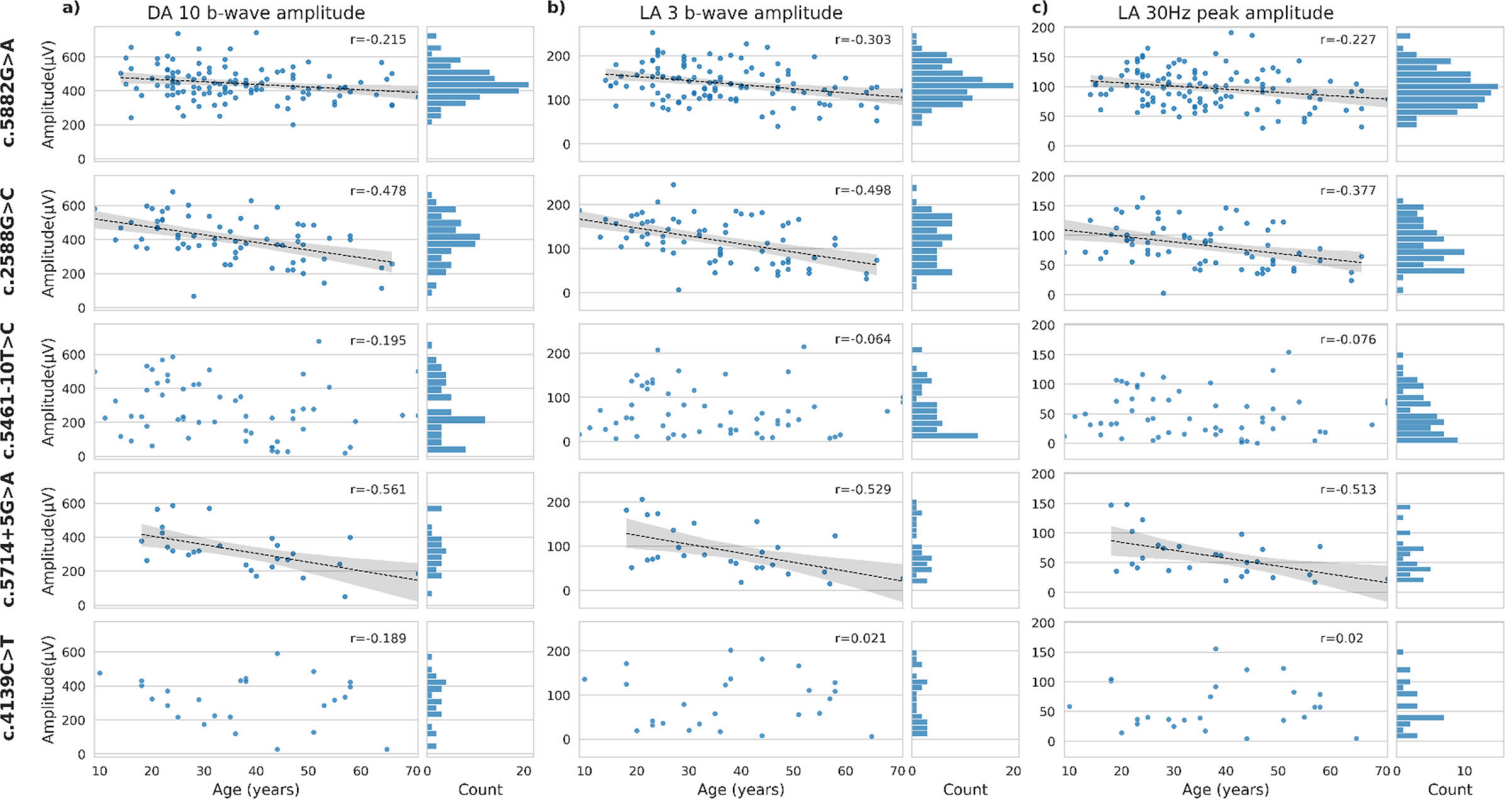
Amplitudes of ERG components plotted against age and illustrating the data associated with the commonest genetic variants, displayed in descending order from the most common (c.5882G>A; prevalence 22.55%) to the fifth most common (c.4139C>T) variant (*left-hand plots*). Linear regression lines are shown to indicate significant correlation with age (*P* < 0.05); *shaded areas* represent confidence limits. Histograms illustrate parameter distributions (*right-hand plots*).

The distribution of ERG components and variation with age were further investigated for the five most prevalent *ABCA4* variants (Fig. 4). Three of the top five variants were associated with significant (Pearson correlation *P* < 0.05) negative gradients for DA 10 b-wave amplitudes, LA 3 b-wave amplitudes, and LA 30 Hz flicker peak amplitudes, consistent with age-associated loss of cone and rod system function (Fig. 3, rows 1, 2, and 4). Plots for the DA 10 and LA 3 ERG a-wave amplitudes and LA 30 Hz ERG peak times are shown in Supplementary Figure S1. Annual rates of ERG decline for all ERG components are given in Supplementary Table S3.

### Classification of ERG Phenotype

An ensemble of machine learning models was used to classify ERG phenotypes into groups 1 to 3, based on the DA 10, LA 3, and LA 30 Hz ERGs and the results compared with the expert analysis. In a repeated fivefold nested cross-validation within the ERG data set, the overall holdout test group accuracy was 91.8% (SE, 0.17%), with an average accuracy of 96.7%, 39.3%, and 93.8% for ERG phenotype groups 1, 2, and 3, respectively, and an average κ value of 0.84. The normalized confusion matrix for each group is displayed in Table 2^3^ (see Supplementary Fig. S2a for one-versus-all receiver operating characteristic [ROC] curve).

**Table 2.** Normalized Confusion Matrix for ERG Phenotype Classification into One of Three Groups, According to Repeated Nested Cross-Validation Holdout Test Sets

	Model Phenotype Prediction		
Expert-Assigned Phenotype	Group 1	Group 2	Group 3
Group 1	0.97	0.015	0.013
Group 2	0.47	0.39	0.19
Group 3	0.036	0.021	0.94

**Table 3.** Normalized Confusion Matrix for Binary Classification into Restricted (Mild) and Generalized (Severe) ERG Phenotypes, According to Repeated Nested Cross-Validation Holdout Test Sets

	Model Phenotype Prediction	
Expert-Assigned Phenotype	Restricted Disease	Generalized Disease
Restricted disease (group 1)	0.95	0.047
Generalized disease (groups 2 and 3)	0.087	0.91

Groups 2 and 3 were combined for a binary classification of restricted (group 1) or generalized (groups 2 and 3) disease phenotypes. In a repeated fivefold nested cross-validation, the soft-voting ensemble model achieved an average holdout test group accuracy of 93.6% (SE, 0.14%), a sensitivity of 0.91, a specificity of 0.95, and a mean area under the curve (AUC) of 0.93 (ROC curve in Supplementary Fig. S2b) for generalized disease. The normalized confusion matrix for each group is displayed in Table 3.

**Figure 5.**
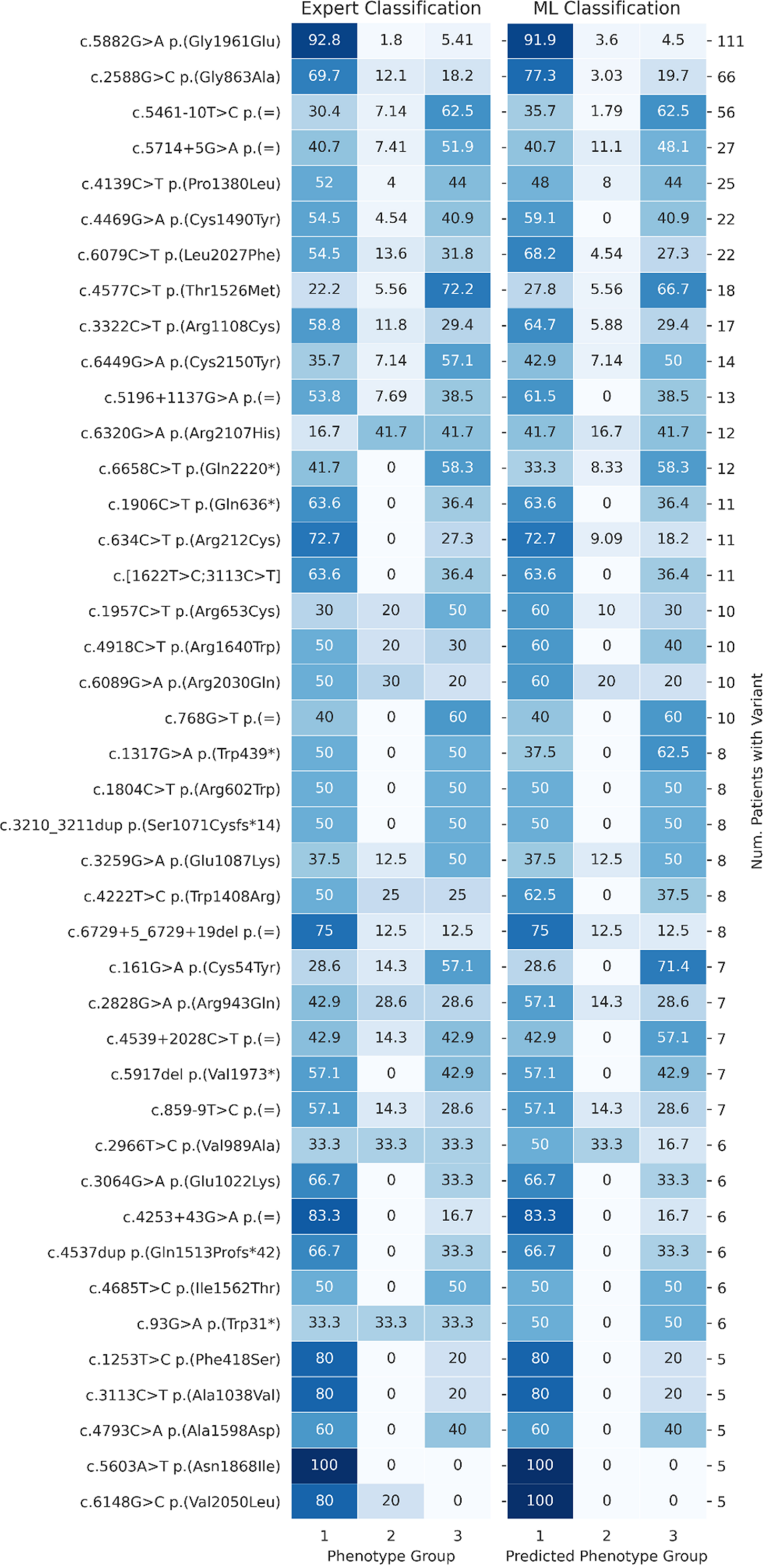
The percentage of patients falling into each ERG phenotype group is included for the 42 most frequent *ABCA4* variants, according to expert analysis (*left*) and the machine learning method (*right*). Each row relates to all the patients with a particular variant. The color coding allows appreciation of the similarities and level of concordance between the two methods (high to low values are highlighted by *dark* to *light shading*).

Figure 5 displays the expert classification and the machine learning phenotype prediction for each of the most prevalent variants (present in at least five patients), highlighting concordance between methods.

### Quantification of Variant Severity

**Figure 6.**
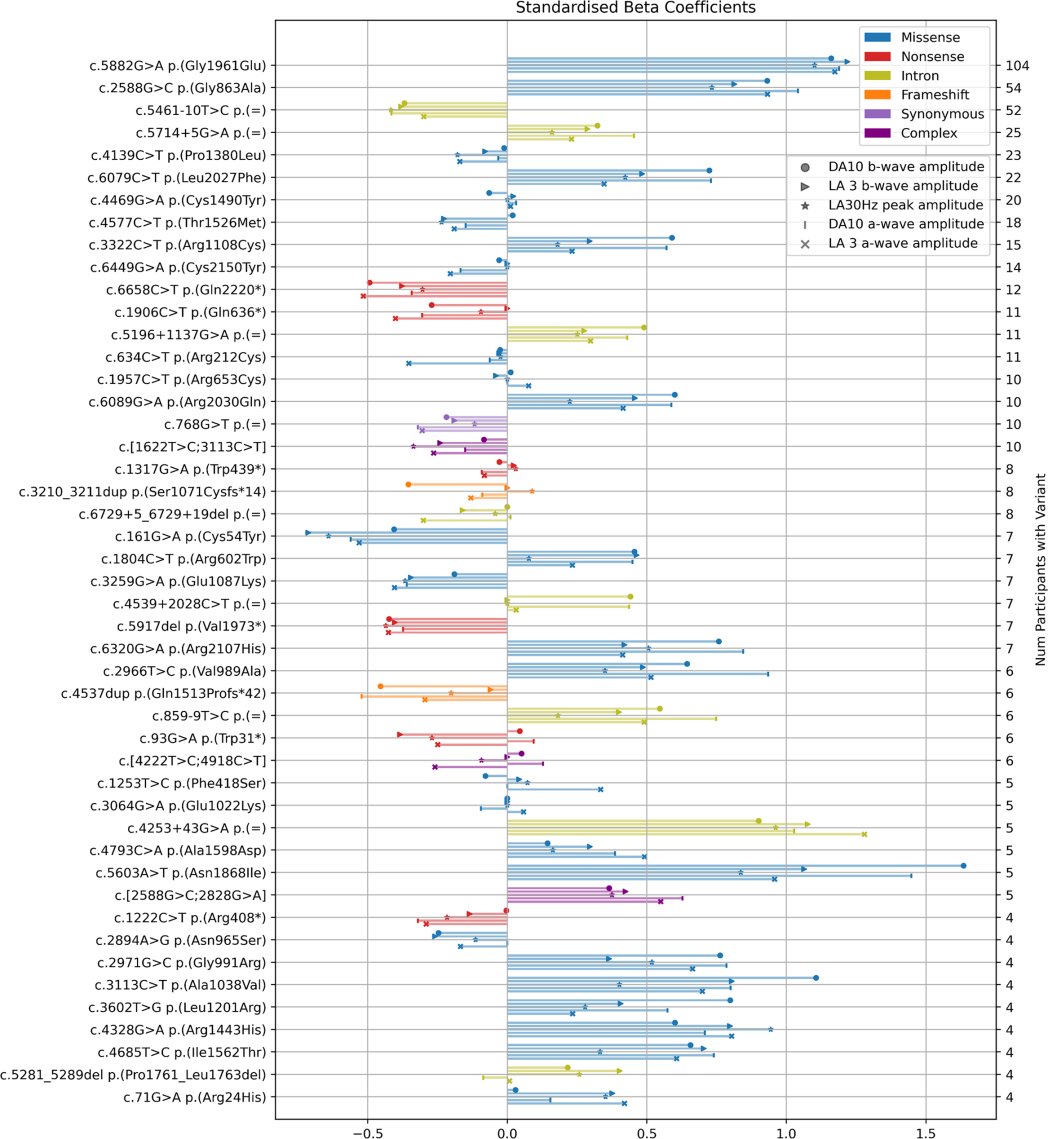
Standardized elastic net regression β-coefficients displayed for the 47 most commonly occurring variants (in combination with at least one other known variant), for prediction of DA 10 b-wave amplitude (●), LA 3 b-wave amplitude (▸), and LA 30 Hz flicker peak amplitude (★). Absolute DA10 a-wave amplitude (l) and absolute LA 3 a-wave amplitude (x).

The amplitudes of the DA 10 and LA 3 a-waves and b-waves and LA 30 Hz ERG were predicted for the genetic variants of study participants (in whom biallelic variants had been found) using an elastic net regression model. In a leave-one-out cross-validation detailed in Supplementary Figure S3, regression against automatically measured ERG component amplitudes resulted in an average *r*^2^ = 0.288, with *r*^2^ = 0.325 and *r*^2^ = 0.320 for DA 10 a-waves and b-waves, respectively. Figure 6 displays the standardized β-coefficient values for the 47 most common genetic variants in the cohort. Regression analysis showed higher coefficient values are associated with the typically milder variants c.5882G>A p.(Gly1961Glu),^18^^,^^19^ c.5603A>T p.(Asn1868Ile),^20^ and intronic variant c.4253+43G>A,^21^ while lower coefficient values were associated with known nonsense and frameshift variants, as well as known null-like intronic variant c.5461-10T>C.^22^^,^^23^ Several missense mutations also displayed more negative coefficients, suggesting a more deleterious effect on *ABCA4* protein function. Supplementary Table S4 contains the standardized β-coefficients for the 101 variants occurring in two or more patients. For each variant, β-coefficients were similar for all ERG components analyzed (average correlation between ERG parameters *r* = 0.82).

## Discussion

This study investigates a uniquely large single-center cohort of patients with *ABCA4*-related macular and/or retinal dysfunction, characterized using international standard ERG protocols. Different ERG phenotypes were established across an age range of more than seven decades and relationships to genotypes examined, through application of supervised machine learning to ERG data and genotype–phenotype relationships.

The study involved retrospective interrogation of legacy data obtained with different instruments but using consistent ERG protocols and considered factors such as pupil size, technical quality of recordings, and sex differences. The study highlights the value of adopting standard (ISCEV) ERG techniques and demonstrates methods of optimizing data for automated interrogation, pertinent to future studies and the possibility of pooling of multicenter electrophysiologic data.

There was a high degree of interocular symmetry of ERG amplitude parameters. This is a feature of most inherited retinal diseases and may be of diagnostic relevance in cases of suspected Stargardt disease, particularly if genetic confirmation is lacking. An inherent feature of visual electrophysiology is the presence of physiologic noise and artifacts (e.g., due to muscle activity or eye movements). Noise is ideally minimized at source and binocular recordings usually important for diagnosis, but averaging between eyes may improve the signal-to-noise ratio, justified in the current study by the high level of interocular symmetry and allowing full utilization of available legacy data.

### Prevalence of Different Groups and the Spectrum of Disease

The majority of patients fell into group 1, with almost 35% in group 3 and a small number in group 2 (7.4%). Eighty percent of patients with a group 1 ERG phenotype have previously been shown to have stable ERGs at 10-year follow-up, whereas a large proportion in group 2 progress in time to group 3.^6^ This may explain the rarity of group 2 patients. Although the discriminating feature between groups 2 and 3 is the presence of generalized rod system dysfunction, our findings show that cone system function (LA ERGs in Fig. 3) also differs between the two groups, consistent with different stages of the same progressive subtype. The distribution of parameters over the cohort (Figs. 3d–m) suggests a continuous spectrum of disease, with little evidence of multiple separate peaks or modal ranges to indicate distinct subgroups.

In cross-sectional studies of healthy individuals, ERG amplitudes generally decline with age.^24^ This also occurred in our patient cohort, particularly in groups 1 and 2. Paradoxically, some ERG components in group 3 appeared to become larger with increasing age (Fig. 3), but this is likely explained by younger patients in group 3 tending to have more aggressive and severe disease than those presenting later in life. This highlights a need for caution when deriving estimates of age-related effects from cross-sectional rather than longitudinal data.

### Parameter Distributions and Variation With Age for the Five Most Common Variants

The diverse range of *ABCA4* genetic variants that can constitute a person's genotype is likely to underlie much of the variability in phenotype, although *trans*-acting genetic and environmental modifiers might also exist. To investigate this, the range of ERG values was investigated in cases harboring one of the five most prevalent variants in our cohort (Fig. 4). The commonest variant (top panels of Fig. 4) was associated with a relatively narrow parameter distribution with a peak (highest counts) in the normal range, consistent with this being a mild variant: regardless of the variant on the other allele (whether it mildly or severely impairs protein production or function), dysfunction is restricted to the macula, and the normal ERGs show an age-associated decline similar to that seen in healthy cohorts.^24^ A broader distribution or multiple peaks, including high frequencies of low ERG amplitudes (see, e.g., third row in Fig. 4), is consistent with variants having a more severe impact. When paired with a mild variant on the other allele, disease may be restricted, with normal ERGs, but when paired with severe variants, disease is more severe. This likely contributes to the apparent lack of age-related ERG decline associated with some of the more severe variants (Fig. 4). Small numbers will limit the reliability of this analysis.

### Application of Machine Learning for Automated Group Classification

Automated group classification using a voting ensemble showed high concordance with expert classification for groups 1 and 3, which constituted 92.6% of the cohort. Overall accuracy was 91.8% with high specificity and sensitivity for groups 1 and 3. Sensitivity and specificity for group 2 were low, possibly reflecting the low number of cases (and hence far fewer cases available for training). When groups 2 and 3 were combined, based on the rationale that they reflect earlier and later time points in a common disease trajectory, accuracy was again high (93.6%), with high sensitivity and specificity for both classes (high AUC of 0.93). As the groups have prognostic significance (particularly group 1 compared with groups 2 and 3), this provides proof of principle that automated machine learning classification has great potential in this condition.

Voting ensembles can improve variance without increasing model bias by averaging out the diversity in the different base models. In preliminary experiments, the voting ensemble was found to increase accuracy over the results of individual classifiers. Given the modular nature of the model architecture, it is also adaptable to the variation in numbers of ERG traces per person and allows inference where data were only available from single eyes, without requiring missing data imputation.

### Expert and Machine Learning Group Classification for the 42 Commonest Variants

The proportion of cases falling into each group for each of the 42 commonest variants (each present in five or more patients) was quantified, according to expert and automated classification (Fig. 5). Cases harboring milder variants will almost all confer a group 1 phenotype, irrespective of the variant on the other allele, while the more severe variants will show high proportions in both groups 1 and 3 (depending on whether they are paired with a mild or severe variant, respectively). Both expert and machine learning–derived classifications show similar patterns by variant. A notable exception is the c.6320G>A p.(Arg2107His) variant: most of these cases were expert-classified as group 2 or 3, but the more prevalent groups by machine learning were groups 1 and 3. The reason is uncertain, but this outlier may relate to only 12 cases being available.

### Regression-Based Quantification of Variant Severity

An elastic net regression approach was additionally used to quantify severity of the top 47 variants by assessing effects on ERG components, independent of any consideration of ERG group. The results in Figure 6 are color-coded by type of variant. Those with more positive coefficients (lines extending to symbols on the right of the chart) denote variants that have less effect on ERG amplitudes (thus predisposing to more restricted disease), while those with less positive or more negative coefficients (symbols on the left of the chart) predispose to more generalized disease. A striking feature is that the standardized coefficients are similar for each ERG parameter (average *r* = 0.82), indicating consistency across the different stimulus responses for a given variant. The results are also highly consistent with what is already known about specific milder variants, such as c.5882G>A p.(Gly1961Glu),^18^^,^^19^ c.5603A>T p.(Asn1868Ile),^20^ and c.4253+43G>A,^21^ and nonsense and frameshift variants, including c.5461-10T>C,^22^^,^^23^ supporting the accuracy of this novel approach.

Previous studies have used different approaches to quantify variant severity and derived metrics relating to delay in disease initiation (based on perimetric or Optical Coherence Tomography (OCT) ellipsoid zone data).^25^^,^^26^ We found significant positive correlation between our ERG-based scoring of variant severity and these metrics (*r* = 0.639 and *r* = 0.668 for correlation between our scores and the metrics of Pfau et al.^25^ and Cideciyan et al.,^26^ respectively). These correlations are depicted in Supplementary Figure S4.

The strength of our approach is limited for the less common variants but may prove fruitful in future studies of larger cohorts pooled from multiple centers. In future, such approaches may allow a prediction, based on genotype, as to whether an individual will develop disease restricted to the macula or more widespread panretinal disease (with much greater impact on quality of life). This would allow more accurate prognostic advice and also inform the timing and suitability for novel therapeutic approaches currently in clinical trials.

### Study Limitations and Future Directions

ERG data in their nature are instrument dependent, prone to noise artifacts, and are relatively scarce outside of specialist settings, presenting challenges for training generalizable machine learning models. Imbalance in ERG phenotypic groups within our *ABCA4* retinopathy cohort and diversity in genetic background may also impact generalizability through the potential for overfitting on small subpopulations. Additionally, the current study is limited to the analysis of ISCEV standard ERGs recorded with gold foil electrodes at a single visit, potentially restricting applicability to other ERG methods. Longitudinal data could further establish the prognostic significance of the ERG phenotype and are likely to be similarly amenable to machine learning investigation. It would also be interesting to extend our analysis to a larger ERG data set involving different pathologies and to develop models through unsupervised learning suited to identifying patterns in large and more complex data sets, including other types of visual electrophysiology. There is potential, using such techniques, to identify hitherto undefined features inherent within the waveforms that may have clinical significance.

## Conclusion

Visual electrophysiology has long offered valuable insights into retinal and visual pathway health and disease, but interpretation requires significant expertise and resources. Standardization of recording methods has facilitated meaningful interlaboratory comparisons and pooling of data, but the international standard ERG analysis involves assessment of relatively few components, and it is tempting to speculate that some clinically meaningful ERG characteristics may be revealed by further automated analysis. The use of artificial intelligence is not new in health care, but its application to full-field ERGs in inherited retinal diseases is unexplored, and to the best of our knowledge, this is the first study to demonstrate applicability of machine learning directly to full-field ERG analysis in *ABCA4* retinopathy.

## Data Sharing

Deidentified data used in this study are not publicly available at present. Parties interested in data access should contact Anthony Robson (ERG data; anthony.robson3@nhs.net) or Omar Mahroo (o.mahroo@nhs.net). Applications will need to undergo ethical and legal approvals by Moorfields Eye Hospital NHS Foundation Trust.

## Supplementary Material

Supplement 1

Supplement 2
