# Supplementary material for: Phenotyping of ABCA4 Retinopathy by Machine Learning Analysis of Full-Field Electroretinography
Source: Transl Vis Sci Technol. 2022 Sep 30;11(9):34. doi: 10.1167/tvst.11.9.34 (PMC9527330; doi:10.1167/tvst.11.9.34)

Supplementary Figures

Supplementary Figure 1. ERG component variation over the five most frequent *ABCA4* variants for ERG components DA 10 a-wave, LA 3 a-wave, and LA 30Hz peak time.

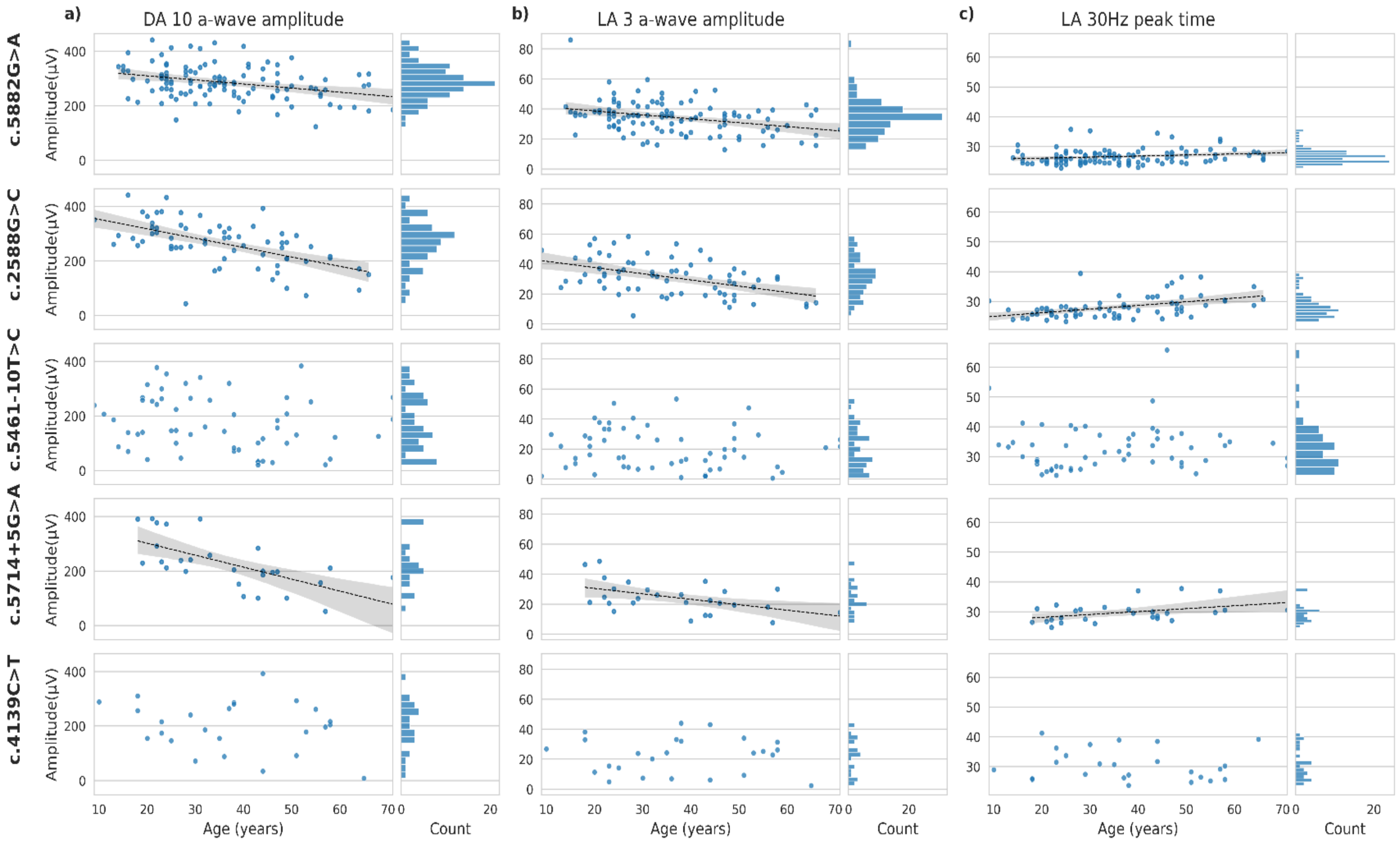

Supplementary Figure 2. ROC curves for a) one-vs-all three class classification for ERG groups 1, 2 and 3 and b) for binary classification of ERG phenotype into generalised (severe) and restricted (mild) disease.

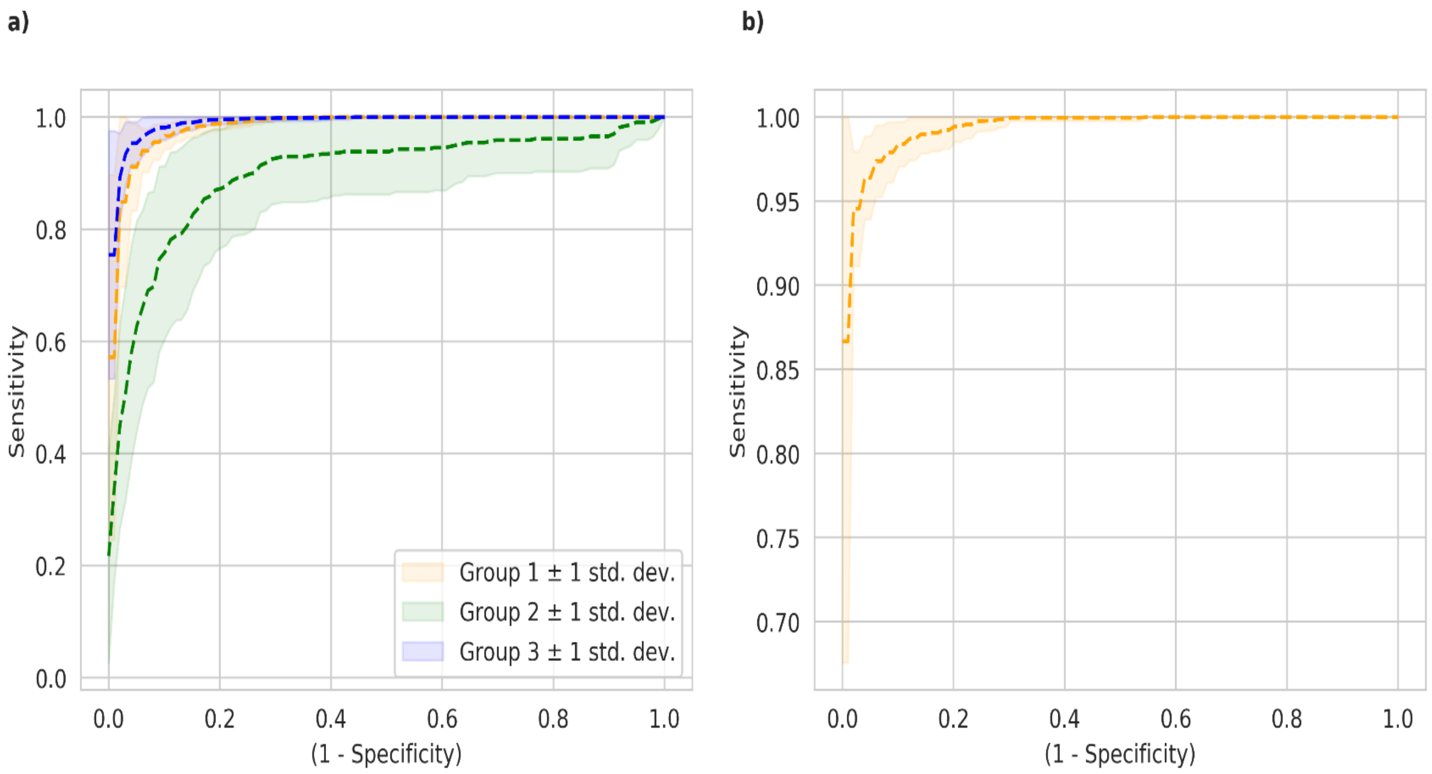

Supplementary Figure 3. Comparison of observed and expected results for the test group of single participant ERG component estimation from a leave-one-out cross validation of variant severity estimation for the five dependent variables included in elastic net regression analysis of genetic variant background a) DA10 b-wave amplitude b) LA 3 b-wave amplitude c) LA30Hz peak amplitude d) DA10 a-wave amplitude and e) LA 3 a-wave amplitude.

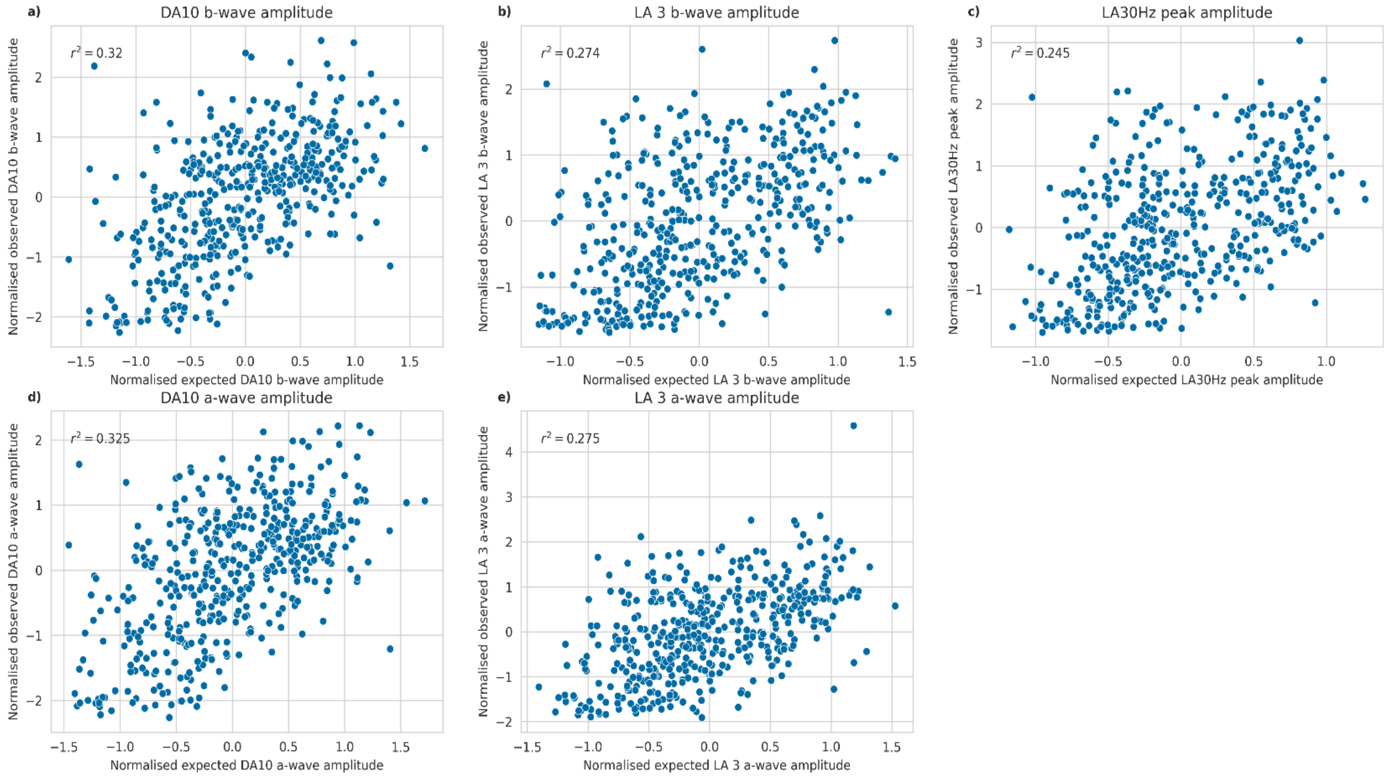

Supplementary Figure 4. Comparison with years delay in disease initiation regression based estimates of variant severity for variants with estimates given in both sets of analysis from a) Pfau *et al.* [25] PMID35076026 and b) Cideciyan *et al.* [26] PMID19074458.

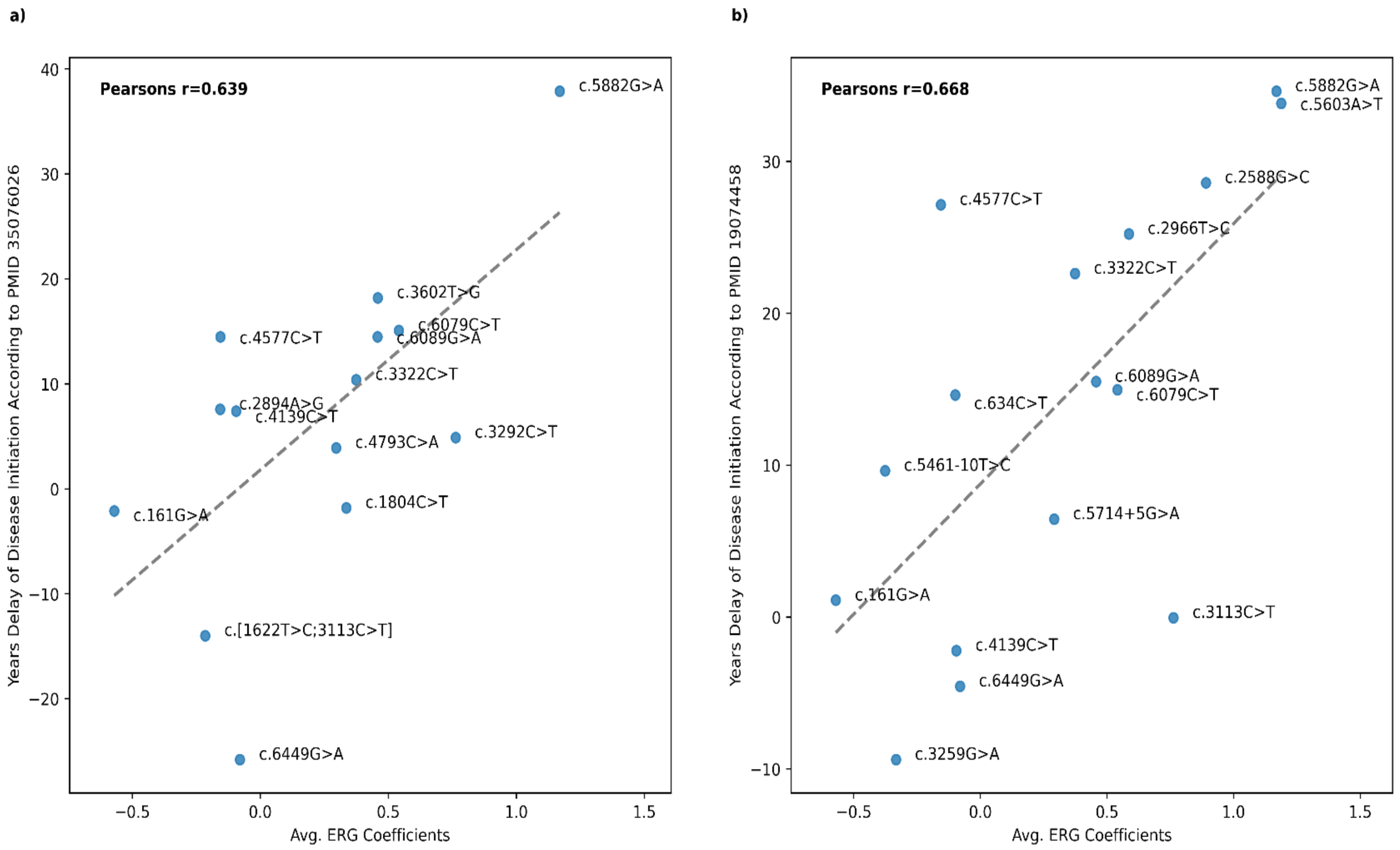

Supplement: Supplement 1 [file tvst-11-9-34_s001.pdf]
