# Supplementary material for: Phenotyping of ABCA4 Retinopathy by Machine Learning Analysis of Full-Field Electroretinography
Source: Transl Vis Sci Technol. 2022 Sep 30;11(9):34. doi: 10.1167/tvst.11.9.34 (PMC9527330; doi:10.1167/tvst.11.9.34)
Supplement: Supplement 2 [file tvst-11-9-34_s002.pdf]

Supplementary Information

Supplementary Tables

Supplementary Table 1. Butterworth filter parameters and time threshold for identification of ERG components.

Stimulus	High pass frequency cut-off	Low pass frequency cut-off	Lower time threshold (ms after flash)
**DA 10 a-wave**	-	0.125	7
**DA 10 b-wave**	0.007	0.0125	20
**LA 3 a-wave**	-	0.1	10
**LA 3 b-wave**	0.0005	0.035	25
**LA 30Hz minima (trough)**	-	0.125	5
**LA 30Hz maxima (peak)**	0.005	0.05	20

Supplementary Table 2. *ABCA4* variant prevalence for study participants with bi-allelic variants.

Coding description	Protein description	Number of participants carrying the variant	Variant prevalence in cohort (%)
c.5882G>A	p.(Gly1961Glu)	111	22.3
c.2588G>C	p.(Gly863Ala)	66	13.3
c.5461-10T>C	p.(=)	56	11.3
c.5714+5G>A	p.(=)	27	5.4
c.4139C>T	p.(Pro1380Leu)	25	5
c.4469G>A	p.(Cys1490Tyr)	22	4.4
c.6079C>T	p.(Leu2027Phe)	22	4.4
c.4577C>T	p.(Thr1526Met)	18	3.6
c.3322C>T	p.(Arg1108Cys)	17	3.4
c.6449G>A	p.(Cys2150Tyr)	14	2.8
c.5196+1137G>A	p.(=)	13	2.6
c.6320G>A	p.(Arg2107His)	12	2.4
c.6658C>T	p.(Gln2220*)	12	2.4
c.1906C>T	p.(Gln636*)	11	2.2
c.634C>T	p.(Arg212Cys)	11	2.2
c.[1622T>C;3113C>T]		11	2.2
c.6089G>A	p.(Arg2030Gln)	10	2
c.4918C>T	p.(Arg1640Trp)	10	2
c.1957C>T	p.(Arg653Cys)	10	2
c.768G>T	p.(=)	10	2
c.3259G>A	p.(Glu1087Lys)	8	1.6
c.6729+5_6729+19del	p.(=)	8	1.6
c.1804C>T	p.(Arg602Trp)	8	1.6

c.4222T>C	p.(Trp1408Arg)	8	1.6
c.1317G>A	p.(Trp439*)	8	1.6
c.3210_3211dup	p.(Ser1071Cysfs*14)	8	1.6
c.2828G>A	p.(Arg943Gln)	7	1.4
c.161G>A	p.(Cys54Tyr)	7	1.4
c.859-9T>C	p.(=)	7	1.4
c.5917del	p.(Val1973*)	7	1.4
c.4539+2028C>T	p.(=)	7	1.4
c.2966T>C	p.(Val989Ala)	6	1.2
c.3064G>A	p.(Glu1022Lys)	6	1.2
c.4253+43G>A	p.(=)	6	1.2
c.4685T>C	p.(Ile1562Thr)	6	1.2
c.4537dup	p.(Gln1513Profs*42)	6	1.2
c.93G>A	p.(Trp31*)	6	1.2
c.6148G>C	p.(Val2050Leu)	5	1
c.5603A>T	p.(Asn1868Ile)	5	1
c.4793C>A	p.(Ala1598Asp)	5	1
c.1253T>C	p.(Phe418Ser)	5	1
c.3113C>T	p.(Ala1038Val)	5	1
c.2971G>C	p.(Gly991Arg)	4	0.8
c.5281_5289del	p.(Pro1761_Leu1763del)	4	0.8
c.3602T>G	p.(Leu1201Arg)	4	0.8
c.2894A>G	p.(Asn965Ser)	4	0.8
c.4328G>A	p.(Arg1443His)	4	0.8
c.71G>A	p.(Arg24His)	4	0.8
c.1222C>T	p.(Arg408*)	4	0.8
c.5196+1G>A	p.(=)	3	0.6
c.666_678del	p.(Lys223Metfs*14)	3	0.6
c.4216C>T	p.(His1406Tyr)	3	0.6
c.5516T>C	p.(Phe1839Ser)	3	0.6

c.4234C>T	p.(Gln1412*)	3	0.6
c.5693G>A	p.(Arg1898His)	3	0.6
c.4253+4C>T	p.(=)	3	0.6
c.2522A>C	p.(Gln841Pro)	3	0.6
c.466A>G	p.(Ile156Val)	3	0.6
c.1648G>A	p.(Gly550Arg)	3	0.6
c.3056C>T	p.(Thr1019Met)	3	0.6
c.5908C>T	p.(Leu1970Phe)	3	0.6
c.4363T>C	p.(Cys1455Arg)	3	0.6
c.454C>T	p.(Arg152*)	3	0.6
c.3292C>T	p.(Arg1098Cys)	3	0.6
c.6088C>T	p.(Arg2030*)	3	0.6
c.5161_5162del	p.(Thr1721Hisfs*65)	3	0.6
c.6118C>T	p.(Arg2040*)	3	0.6
c.1715G>A	p.(Arg572Gln)	3	0.6
c.6286G>A	p.(Glu2096Lys)	3	0.6
c.2813T>C	p.(Phe938Ser)	3	0.6
c.5018+5G>A	p.(=)	2	0.4
c.4919G>A	p.(Arg1640Gln)	2	0.4
c.2560G>A	p.(Ala854Thr)	2	0.4
c.2791G>A	p.(Val931Met)	2	0.4
c.2564G>A	p.(Trp855*)	2	0.4
c.5018+2T>C		2	0.4
c.5088C>G	p.(Ser1696Arg)	2	0.4
c.[5461-10T>C;5603A>T]		2	0.4
c.4739T>C	p.(Leu1580Ser)	2	0.4
c.2912C>A	p.(Thr971Asn)	2	0.4
c.4594G>A	p.(Asp1532Asn)	2	0.4
c.4537del	p.(Gln1513Argfs*13)	2	0.4
c.3081T>G	p.(Tyr1027*)	2	0.4

c.4326C>A	p.(Asn1442Lys)	2	0.4
c.4319T>C	p.(Phe1440Ser)	2	0.4
c.4070C>T	p.(Ala1357Val)	2	0.4
c.3808G>T	p.(Glu1270*)	2	0.4
c.3758C>T	p.(Thr1253Met)	2	0.4
c.3289A>T	p.(Arg1097*)	2	0.4
c.3305A>T	p.(Asp1102Val)	2	0.4
c.331_332del	p.(Glu111Thrfs*49)	2	0.4
c.3386G>T	p.(Arg1129Leu)	2	0.4
c.4463G>T	p.(Cys1488Phe)	2	0.4
c.2345G>A	p.(Trp782*)	2	0.4
c.180del	p.(Met61Cysfs*17)	2	0.4
c.655A>T	p.(Arg219*)	2	0.4
c.5578C>T	p.(Arg1860Trp)	2	0.4
c.5413A>G	p.(Asn1805Asp)	2	0.4
c.2023G>A	p.(Val675Ile)	2	0.4
c.1819G>A	p.(Gly607Arg)	2	0.4
c.6694G>A	p.(Glu2232Lys)	2	0.4
c.1726G>C	p.(Asp576His)	2	0.4
c.1140T>A	p.(Asn380Lys)	2	0.4
c.1805G>A	p.(Arg602Gln)	2	0.4
c.1609C>T	p.(Arg537Cys)	2	0.4
c.6352del	p.(Arg2118Glufs*27)	2	0.4
c.2239del	p.(Leu747Cysfs*40)	2	0.4
c.223T>G	p.(Cys75Gly)	2	0.4
c.1648G>T	p.(Gly550*)	2	0.4
c.1903C>A	p.(Gln635Lys)	2	0.4
c.658C>T	p.(Arg220Cys)	2	0.4

c.926C>G	p.(Pro309Arg)	2	0.4
c.1335C>G	p.(Ser445Arg)	2	0.4
c.1460G>A	p.(Arg487Gln)	1	0.2
c.3197T>G	p.(Met1066Arg)	1	0.2
c.2382G>A	p.(=)	1	0.2
c.1531C>T	p.(Arg511Cys)	1	0.2
c.3178C>T	p.(Gln1060*)	1	0.2
c.1557C>A	p.(Cys519*)	1	0.2
c.1622T>C	p.(Leu541Pro)	1	0.2
c.3091A>T	p.(Lys1031*)	1	0.2
c.1654G>A	p.(Val552Ile)	1	0.2
c.1433T>C	p.(Ile478Thr)	1	0.2
c.3212C>T	p.(Ser1071Leu)	1	0.2
c.1343T>A	p.(Met448Lys)	1	0.2
c.3262C>A	p.(Pro1088Thr)	1	0.2
c.1018T>C	p.(Tyr340His)	1	0.2
c.1019A>C	p.(Tyr340Ser)	1	0.2
c.1037A>C	p.(Lys346Thr)	1	0.2
c.3303G>A	p.(Trp1101*)	1	0.2
c.3299T>A	p.(Ile1100Asn)	1	0.2
c.1069_1075del	p.(Asp357Serfs*15)	1	0.2
c.108del	p.(Leu37Trpfs*3)	1	0.2
c.1196del	p.(Leu399Argfs*13)	1	0.2
c.3210_3211insGT	p.(Ser1071Valfs*14)	1	0.2
c.3260A>G	p.(Glu1087Gly)	1	0.2
c.1292G>A	p.(Trp431*)	1	0.2
c.3259G>T	p.(Glu1087*)	1	0.2
c.3233G>A	p.(Gly1078Glu)	1	0.2
c.3224C>A	p.(Ala1075Asp)	1	0.2

c.3211insGT		1	0.2
c.1339C>T	p.(Gln447*)	1	0.2
c.1681G>A		1	0.2
c.3050+1G>C	p.(=)	1	0.2
c.1749G>C	p.(Lys583Asn)	1	0.2
c.1757A>G	p.(Asp586Gly)	1	0.2
c.2843G>C	p.(Arg948Pro)	1	0.2
c.1964T>G	p.(Phe655Cys)	1	0.2
c.1984dup	p.(Ala662Glyfs*104)	1	0.2
c.2827C>T	p.(Arg943Trp)	1	0.2
c.2041C>T	p.(Arg681*)	1	0.2
c.214G>A	p.(Gly72Arg)	1	0.2
c.2713del	p.(Glu905Argfs*27)	1	0.2
c.2617T>C	p.(Phe873Leu)	1	0.2
c.2160+1G>C		1	0.2
c.2588G>A	p.(Gly863Glu)	1	0.2
c.2587+2T>C	p.(=)	1	0.2
c.21dup	p.(Gln8Thrfs*46)	1	0.2
c.2568C>A	p.(Tyr856*)	1	0.2
c.2560G>T	p.(Ala854Ser)	1	0.2
c.2265C>G	p.(Phe755Leu)	1	0.2
c.2297G>A	p.(Gly766Asp)	1	0.2
c.3329-1G>A	p.(=)	1	0.2
c.1938-1G>A	p.(=)	1	0.2
c.2861A>C	p.(Tyr954Ser)	1	0.2
c.286A>G	p.(Asn96Asp)	1	0.2
c.1817G>A	p.(Gly606Asp)	1	0.2
c.1760+1G>A	p.(=)	1	0.2
c.3050+34C>T		1	0.2
c.2510T>C	p.(Leu837Pro)	1	0.2

c.1760+3G>C		1	0.2
c.1760G>A	p.(Arg587Lys)	1	0.2
c.302+5G>C		1	0.2
c.2972G>T	p.(Gly991Val)	1	0.2
c.1856T>A	p.(Ile619Asn)	1	0.2
c.1937+1G>A	p.(=)	1	0.2
c.1874_1912del	p.(Gln625_Met637del)	1	0.2
c.293A>G	p.(Asn98Ser)	1	0.2
c.2930C>T	p.(Thr977Met)	1	0.2
c.1913C>T	p.(Pro638Leu)	1	0.2
c.2915C>A	p.(Thr972Asn)	1	0.2
c.1917C>A	p.(Tyr639*)	1	0.2
c.1928T>G	p.(Val643Gly)	1	0.2
c.3329-17T>G		1	0.2
c.4253+5G>T	p.(=)	1	0.2
c.*55G>T	p.(=)	1	0.2
c.5196+1216C>A	p.(=)	1	0.2
c.5933_5943dup	p.(Phe1982Lysfs*14)	1	0.2
c.5932A>G	p.(Lys1978Glu)	1	0.2
c.5929G>A	p.(Gly1977Ser)	1	0.2
c.5904del	p.(Phe1968Leufs*6)	1	0.2
c.5899-2del	p.(=)	1	0.2
c.5898+1G>A	p.(=)	1	0.2
c.5761G>A	p.(Val1921Met)	1	0.2
c.571-1G>T	p.(=)	1	0.2
c.568+434a>G		1	0.2
c.5645T>C	p.(Met1882Thr)	1	0.2
c.5584G>A	p.(Gly1862Ser)	1	0.2
c.5527C>G	p.(Arg1843Gly)	1	0.2

c.5519G>T	p.(Cys1840Phe)	1	0.2
c.5463G>A	p.(=)	1	0.2
c.53G>A	p.(Arg18Gln)	1	0.2
c.5396A>G	p.(Asn1799Ser)	1	0.2
c.5381C>A	p.(Ala1794Asp)	1	0.2
c.5327C>T	p.(Pro1776Leu)	1	0.2
c.740A>C	p.(Asn247Thr)	1	0.2
c.5315G>A	p.(Trp1772*)	1	0.2
c.763C>T	p.(Arg255Cys)	1	0.2
c.5228G>A		1	0.2
c.5222del	p.(Leu1741Argfs*37)	1	0.2
c.3370G>T	p.(Asp1124Tyr)	1	0.2
c.5196+1G>T	p.(=)	1	0.2
c.6005+1G>T	p.(=)	1	0.2
c.6098T>G	p.(Leu2033Arg)	1	0.2
c.6112C>T	p.(Arg2038Trp)	1	0.2
c.6438C>T	p.(=)	1	0.2
c.719T>G	p.(Ile240Arg)	1	0.2
c.712C>T	p.(Gln238*)	1	0.2
c.6729+4_6729+18delAGTTG		1	0.2
c.6729+4_6729+18del		1	0.2
c.6721C>G	p.(Leu2241Val)	1	0.2
c.6712C>T	p.(Gln2238*)	1	0.2
c.67-2A>G	p.(=)	1	0.2
c.6647C>T	p.(Ala2216Val)	1	0.2
c.656G>C	p.(Arg219Thr)	1	0.2
c.6479+1G>A	p.(=)	1	0.2
c.6445C>T	p.(Arg2149*)	1	0.2
c.6416G>C	p.(Arg2139Pro)	1	0.2
c.6119G>A	p.(Arg2040Gln)	1	0.2

c.6415C>T	p.(Arg2139Trp)	1	0.2
c.6391G>A	p.(Glu2131Lys)	1	0.2
c.6342G>A	p.(=)	1	0.2
c.6329G>A	p.(Trp2110*)	1	0.2
c.6317G>A	p.(Arg2106His)	1	0.2
c.6305A>G	p.(Asp2102Gly)	1	0.2
c.6229C>T	p.(Arg2077Trp)	1	0.2
c.6227A>C	p.(Lys2076Thr)	1	0.2
c.6207C>T	p.(=)	1	0.2
c.619G>A	p.(Glu207Lys)	1	0.2
c.618C>G	p.(Ser206Arg)	1	0.2
c.5196+1217C>T		1	0.2
c.5195+1137G>C		1	0.2
c.735T>G	p.(Tyr245*)	1	0.2
c.5177C>A	p.(Thr1726Asn)	1	0.2
c.443-453T>C		1	0.2
c.4364G>T	p.(Cys1455Phe)	1	0.2
c.4352+1G>A	p.(=)	1	0.2
c.4316G>A	p.(Gly1439Asp)	1	0.2
c.4253+5G>A	p.(=)	1	0.2
c.868C>T	p.(Arg290Trp)	1	0.2
c.4237_4250del114		1	0.2
c.4195G>A	p.(Glu1399Lys)	1	0.2
c.4098dup	p.(Ile1367Hisfs*55)	1	0.2
c.4069G>A	p.(Ala1357Thr)	1	0.2
c.3994C>T	p.(Gln1332*)	1	0.2
c.3898C>T	p.(Arg1300*)	1	0.2
c.3835_3840delTCTGAT		1	0.2
c.3821T>C	p.(Leu1274Pro)	1	0.2
c.3814-3C>A		1	0.2

c.377G>A	p.(Trp126*)	1	0.2
c.3608G>A	p.(Gly1203Glu)	1	0.2
c.3522G>A	p.(=)	1	0.2
c.3482G>A	p.(Arg1161His)	1	0.2
c.3481C>T	p.(Arg1161Cys)	1	0.2
c.3438C>G	p.(Phe1146Leu)	1	0.2
c.3393del	p.(Ile1132Serfs*16)	1	0.2
c.3392_3393delinsG	p.(Ala1131Glyfs*17)	1	0.2
c.3392C>G	p.(Ala1131Gly)	1	0.2
c.3385C>T	p.(Arg1129Cys)	1	0.2
c.4462T>C	p.(Cys1488Arg)	1	0.2
c.4532C>G	p.(Pro1511Arg)	1	0.2
c.4535_4536CC>AA		1	0.2
c.4926C>G	p.(Ser1642Arg)	1	0.2
c.834del	p.(Asp279Ilefs*21)	1	0.2
c.5114G>A	p.(Arg1705Gln)	1	0.2
c.5113C>T	p.(Arg1705Trp)	1	0.2
c.5087G>A	p.(Ser1696Asn)	1	0.2
c.5077G>A	p.(Val1693Ile)	1	0.2
c.5056G>A	p.(Val1686Met)	1	0.2
c.5051T>A	p.(Ile1684Asn)	1	0.2
c.5044_5058del	p.(Val1682_Val1686del)	1	0.2
c.5041_5055delGTGGTTGCCAT		1	0.2
c.4981del	p.(Leu1661*)	1	0.2
c.4948G>T	p.(Glu1650*)	1	0.2
c.4849G>A	p.(Val1617Met)	1	0.2
c.4538A>G	p.(Gln1513Arg)	1	0.2

c.4775G>A	p.(Gly1592Asp)	1	0.2
c.4771G>A	p.(Gly1591Arg)	1	0.2
c.4727T>G	p.(Leu1576Arg)	1	0.2
c.4722del	p.(Ala1575Hisfs*6)	1	0.2
c.4715C>T	p.(Thr1572Met)	1	0.2
c.4637T>G	p.(Leu1546*)	1	0.2
c.4601del	p.(Leu1534Trpfs*2)	1	0.2
c.4594G>T	p.(Asp1532Tyr)	1	0.2
c.455G>A	p.(Arg152Gln)	1	0.2
c.4539+2066C>T		1	0.2
c.858+1G>T	p.(=)	1	0.2
c.731T>C	p.(Leu244Pro)	1	0.2

Supplementary Table 3. The mean rates of annual amplitude change for participants with at least one copy of the five most frequent *ABCA4* variants in the cohort. ND; no change detected.

**ERG component**	**Rate of ERG component amplitude change (μV/year)**				
	**c.5882G>A**	**c.2588G>C**	**c.5461-10T>C**	**c.5714 +5G>A**	**c.4139 C>T**
**DA 10 b-wave**	-1.54	-4.47	ND	-5.12	ND
**LA 3 b-wave**	-0.92	-1.82	ND	-2.03	ND
**LA 30 Hz Peak Amplitude**	-0.54	-0.97	ND	-1.34	ND
**DA 10 a-wave**	-1.5	-3.44	ND	-4.38	ND
**LA 3 a-wave**	-0.26	-0.41	ND	-0.36	ND

Supplementary Table 4. Standardised beta coefficients the 101 most frequent *ABCA4* variants from elastic net regression with ERG components.

	DA10 b-wave amplitude	LA 3 b-wave amplitude	LA30Hz peak amplitude	DA10 a-wave amplitude	LA 3 a-wave amplitude
c.5882G>A	1.16	1.22	1.1	1.19	1.17
c.2588G>C	0.93	0.81	0.73	1.04	0.93
c.5461-10T>C	-0.37	-0.38	-0.42	-0.42	-0.3
c.5714+5G>A	0.32	0.29	0.16	0.45	0.23
c.4139C>T	-0.01	-0.08	-0.18	-0.03	-0.17
c.6079C>T	0.72	0.48	0.42	0.73	0.35
c.4469G>A	-0.07	0.02	0	0.03	0.01
c.4577C>T	0.02	-0.23	-0.24	-0.15	-0.19
c.3322C>T	0.59	0.3	0.18	0.57	0.23
c.6449G>A	-0.03	0	0	-0.17	-0.2
c.6658C>T	-0.49	-0.38	-0.3	-0.34	-0.52
c.1906C>T	-0.27	0	-0.09	-0.31	-0.4
c.5196+1137G>A	0.49	0.28	0.25	0.43	0.3
c.634C>T	-0.03	-0.03	-0.02	-0.06	-0.35
c.1957C>T	0.01	-0.04	0	0	0.08

c.6089G>A	0.6	0.46	0.22	0.59	0.42
c.768G>T	-0.22	-0.19	-0.12	-0.32	-0.31
c.[1622T>C;3113C>T]	-0.08	-0.24	-0.34	-0.15	-0.26
c.1317G>A	-0.03	0.02	0.03	-0.09	-0.08
c.3210_3211dup	-0.35	0	0.09	-0.09	-0.13
c.6729+5_6729+19del	0	-0.16	-0.04	0.01	-0.3
c.161G>A	-0.41	-0.71	-0.64	-0.56	-0.53
c.1804C>T	0.46	0.46	0.08	0.45	0.23
c.3259G>A	-0.19	-0.35	-0.37	-0.36	-0.4
c.4539+2028C>T	0.44	0	0	0.44	0.03
c.5917del	-0.42	-0.4	-0.44	-0.37	-0.43
c.6320G>A	0.76	0.42	0.51	0.85	0.41
c.2966T>C	0.64	0.49	0.35	0.93	0.51
c.4537dup	-0.45	-0.06	-0.2	-0.52	-0.3
c.859-9T>C	0.55	0.4	0.18	0.75	0.49
c.93G>A	0.04	-0.38	-0.27	0.09	-0.25

c.[4222T>C;4918C>T]	0.05	0	-0.09	0.13	-0.26
c.1253T>C	-0.08	0.04	0.07	0	0.33
c.3064G>A	0	0	0	-0.09	0.06
c.4253+43G>A	0.9	1.08	0.96	1.03	1.28
c.4793C>A	0.14	0.3	0.16	0.39	0.49
c.5603A>T	1.63	1.06	0.84	1.45	0.96
c.[2588G>C;2828G>A]	0.37	0.42	0.38	0.63	0.55
c.1222C>T	0	-0.14	-0.22	-0.32	-0.29
c.2894A>G	-0.25	-0.26	-0.11	0	-0.17
c.2971G>C	0.76	0.36	0.52	0.78	0.66
c.3113C>T	1.11	0.8	0.4	0.8	0.7
c.3602T>G	0.8	0.41	0.28	0.57	0.23
c.4328G>A	0.6	0.8	0.94	0.71	0.8
c.4685T>C	0.66	0.7	0.33	0.74	0.61
c.5281_5289del	0.22	0.4	0.26	-0.09	0.01
c.71G>A	0.03	0.38	0.35	0.15	0.42

c.1648G>A	0.37	0.09	0.23	0.37	0.33
c.2522A>C	-0.02	-0.35	-0.25	-0.4	-0.45
c.2813T>C	0.28	0.4	0.28	0.38	0.33
c.3056C>T	0	0.18	0.11	0	0.09
c.3292C>T	0.88	0.85	0.67	0.73	0.68
c.454C>T	-0.62	-0.66	-0.65	-0.48	-0.73
c.5161_5162del	0.05	0.08	0	0	0.31
c.5196+1G>A	0	0.1	0.27	0.05	0.49
c.6088C>T	0.07	-0.1	0	0	-0.21
c.6118C>T	-0.55	-0.15	-0.08	-0.32	-0.27
c.666_678del	-0.46	-0.63	-0.59	-0.57	-0.48
c.1140T>A	-0.47	-0.12	0	-0.16	-0.24
c.1335C>G	0	0	0	-0.01	0
c.1648G>T	-0.41	-0.06	0	-0.27	-0.12
c.1726G>C	0.19	0	0	0.38	0.19
c.1805G>A	0.42	0.43	0.26	0.58	0.06
c.180del	0.28	0	0	0	0.1
c.1903C>A	-0.29	-0.39	-0.5	-0.21	-0.6

c.2023G>A	0.15	0	0	0.5	0.16
c.2239del	-0.79	-0.26	-0.26	-0.71	-0.5
c.223T>G	0.01	0.12	0.11	0.25	0.19
c.2560G>A	0.53	0.68	0.61	0.52	0.62
c.2564G>A	0	-0.23	0.18	0	-0.28
c.2791G>A	-0.04	-0.14	-0.16	-0.16	-0.35
c.2912C>A	-0.28	-0.32	-0.27	-0.02	-0.24
c.3081T>G	-0.9	-0.86	-0.71	-0.98	-0.8
c.3289A>T	-0.24	-0.39	-0.18	-0.34	-0.41
c.3305A>T	0.52	0.09	0.25	0.5	0.35
c.331_332del	0.6	-0.17	-0.15	0.07	-0.44
c.3386G>T	0	0	0	0.05	0.22
c.3808G>T	0.18	0	0	0.33	-0.08
c.4070C>T	0	0.05	0	-0.22	0.18
c.4234C>T	-0.38	-0.22	-0.18	-0.23	-0.36
c.4253+4C>T	0	0	-0.01	0	-0.08
c.4319T>C	0	0	0	0	0.16
c.4326C>A	0	-0.14	-0.01	-0.29	-0.23
c.4463G>T	0	0.42	0.36	0.21	0.26

c.4537del	0	0	0.11	0.14	0.44
c.4594G>A	0.4	0.49	0.3	0.62	0.7
c.4739T>C	0.65	0.66	0.34	0.82	0.62
c.4918C>T	0.02	0.23	0.39	0.1	0.34
c.4919G>A	-0.94	-0.56	-0.48	-1.03	-0.6
c.5018+2T>C	-0.29	-0.35	-0.13	-0.21	-0.61
c.5088C>G	0.01	0.12	0.11	0.25	0.19
c.5413A>G	0	-0.05	0	0.28	0.18
c.5578C>T	1.3	0.87	0.28	1.06	0.74
c.5908C>T	0.49	0.19	0.27	0.53	0.39
c.6148G>C	0.08	0.09	0	0	0.02
c.6286G>A	0	-0.08	0	0	-0.4
c.6352del	0.61	0.69	0.76	0.62	0.72
c.655A>T	0.31	0.46	0.23	0	-0.1
c.658C>T	-0.15	-0.25	-0.15	-0.22	-0.38
c.[1715G>A;2588G>C]	0	0.02	0.03	0	0.17
c.[5461-10T>C;5603A>T]	0.67	0.39	0.32	0.66	0.52
